# Parliament and the Profession

**Published:** 1918-03-23

**Authors:** 


### PARLIAMENT AND THE PROFESSION.

### Tuberculous Soldiers.

Major Davies asked the Minister of National Service whether his attention had been called to cases of men who have been enlisted into the Army while suffering from tuberculosis, and whose certificates from the tuberculosis officer were ignored by the Medical Boards. Sir Auckland Geddes said he was not aware of such cases. Certificates from tuberculosis officers are not ignored by National Service Medical Boards. All evidence is carefully considered, but it must be obvious that the decision in any case in which the presence of tuberculosis is alleged must rest with the Medical Board who are responsible for the diagnosis.

### Medical Aid for Soldiers' Widows.

Major Davies asked the Pensions Minister whether the scheme for the provision of medical aid for the widows and children of soldiers had been put into operation, and if its scope could not be extended to include the wives and children of soldiers now serving. Sir A. Griffith-Boscawen said that the Minister was allowed by Royal Warrant "to deduct from any pension or allowance under Part II. of the Warrant the cost of any benefit which it may hereafter be decided to substitute for any part of the pension or allowance." He did not favourably view the system of deduction, and the matter is for the present in abeyance.

### Nurses and Chevrons.

Mr. Macpherson stated that it has been decided that medical *personnel*, including nurses serving on hospital ships and ambulance transports, are eligible for chevrons by virtue of such service.

### Small-pox in the East End.

Mr. S. Walsh informed Mr. Anderson that ten cases of small-pox were notified between March 2 and 7 in Stepney, Hackney, Bethnal Green, and East Ham. These cases were removed to hospital, the houses were disinfected, all human contacts are under observation, and all who could be persuaded were vaccinated. It is reported that on March 14 seven further cases have occurred in Stepney amongst the contacts, and have been removed to hospital.

For Nursing Affairs in Ireland, by " rne," see p. 536.

